# The Role of Lipid Membranes in Life’s Origin

**DOI:** 10.3390/life7010005

**Published:** 2017-01-17

**Authors:** David Deamer

**Affiliations:** Department of Biomolecular Engineering, University of California, Santa Cruz, CA 95060, USA; deamer@soe.ucsc.edu; Tel.: +1-831-4595158

**Keywords:** membranes, protocells, encapsulation

## Abstract

At some point in early evolution, life became cellular. Assuming that this step was required for the origin of life, there would necessarily be a pre-existing source of amphihilic compounds capable of assembling into membranous compartments. It is possible to make informed guesses about the properties of such compounds and the conditions most conducive to their self-assembly into boundary structures. The membranes were likely to incorporate mixtures of hydrocarbon derivatives between 10 and 20 carbons in length with carboxylate or hydroxyl head groups. Such compounds can be synthesized by chemical reactions and small amounts were almost certainly present in the prebiotic environment. Membrane assembly occurs most readily in low ionic strength solutions with minimal content of salt and divalent cations, which suggests that cellular life began in fresh water pools associated with volcanic islands rather than submarine hydrothermal vents.

## 1. Introduction

In order to understand the conditions in which the first life emerged, it is important to set the stage in terms of the prebiotic environment. Assuming that life began approximately four billion years ago, the stage has three scales to consider: global, local and microscopic. At the global scale, the Earth’s atmosphere was mostly nitrogen with a substantial fraction of carbon dioxide [1]. There was virtually no oxygen. The crust had cooled sufficiently for water vapor to condense into an ocean [2], but the global temperature was still hot, with one estimate in the range 85–110 °C [3]. Plate tectonics had not yet begun to form large continents. The ocean today is salty and the early ocean was also likely to contain dissolved salts, including divalent alkaline earth cations in the millimolar range.

**Figure 1:**
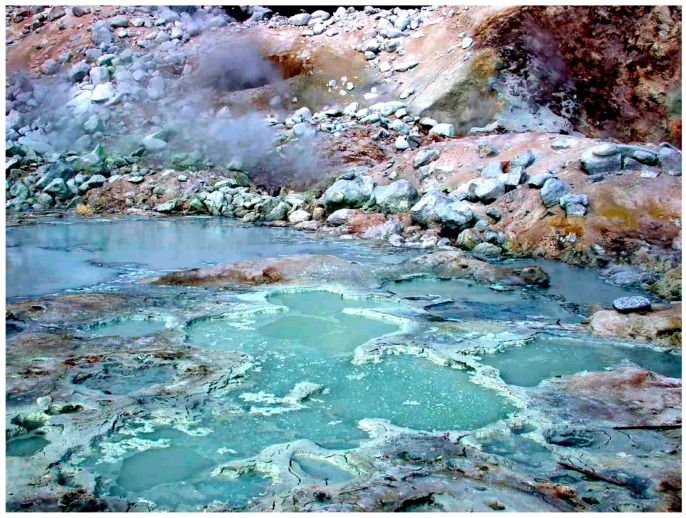
A hydrothermal field on Mount Lassen in northern California.

At the local scale, there is geological evidence that extensive volcanism produced land masses resembling Hawaii and Iceland today [4]. When these emerged from the global ocean, precipitation produced the equivalent of distilled water that accumulated on the volcanic land forms as geysers, hot springs and pools. This is an important point, because it has been generally assumed that life began in the sea, perhaps in submarine hydrothermal vents [5,6,7]. However, an alternative proposition is that cellular life began in hydrothermal fields on volcanic land masses [8,9,10]. Figure 1 shows a hydrothermal field on Mt. Lassen, in northern California, an analogue for similar volcanic sites on the early Earth.

The microscopic scale is represented by interfaces that have specific properties relevant to the origin of life. Hydrothermal vents are characterized by a single interface between minerals and sea water, and the vent minerals have varying degrees of microscopic porosity that may influence the way chemical reactions can occur. In contrast, hydrothermal fields associated with volcanic activity have three interfaces: mineral/water, mineral/atmosphere and water/atmosphere. It is significant that the interfaces are not constant, but instead fluctuate in cycles of hydration and dehydration. The cyclic wet phase is related to precipitation and periodic ebb and flow of water in small pools related to geyser and hot spring activity, while the dry phase is driven by evaporation. As a result of the fluctuating conditions, films of organic solutes become concentrated on mineral surfaces such as glass-like basaltic lava, porous material (pumice, volcanic ash) and clay minerals produced by hydrothermal processing. If some of the organics were amphiphilic compounds, they would self-assemble into vast numbers of microscopic membranous compartments called protocells that encapsulate systems of potential reactants and products [11].

The aim of this review is to consider how the first membranous enclosures could assemble in prebiotic conditions and what role the environment may have played.

Three questions are addressed:

- What amphiphilic compounds might have formed the permeability barrier of primitive cell membranes?
- How were polymers synthesized and then encapsulated to produce protocells?
- Did life begin in a marine or fresh water environment? This question concerns the ability of amphiphilic compounds to assemble into membranes in marine hydrothermal vents compared with hydrothermal fresh water fields.

These questions have also been the focus of research by Pier Luigi Luisi, Jack Szostak, Peter Walde and Pierre-Alain Monnard, whose results provide a foundation for some of the topics discussed here. (For more detailed accounts, see [12,13,14]).

## 2. Properties of Membrane-Forming Amphiphiles in Cells Today

We will begin by briefly describing the main properties of amphiphilic molecules that compose the membranes of contemporary cells, because these properties will constrain the conditions required for self-assembly of membranes from organic compounds available in the environment before life began. An amphiphile is defined as a molecule having both a non-polar hydrocarbon moiety and a polar head group. The simplest amphiphiles are fatty acids having a single hydrocarbon chain and a carboxylic acid head group that is either ionized at alkaline pH ranges (-COO^−^) or protonated (-COOH, neutral) in acidic pH. When the hydrocarbon chain is long enough, 10 carbons or more, the molecules can assemble into monolayers at the air-water interface, micelles in solution, and vesicles having boundaries composed of bimolecular membranes. The membranes are stabilized by the hydrophobic effect [15] and van der Waals interactions. When hydrocarbons are mixed with water, the lowest energy state is for the chains to be excluded from the aqueous phase and form a separate hydrocarbon phase in which van der Waals interactions are maximized.

Phospholipids form the boundaries of compartments within which the most important life processes proceed: metabolism, transport of nutrients and ions, growth by energy-dependent polymerization, storing and transmission of genetic information and replication of nucleic acids. The permeability barrier is provided by the non-polar interior of the bilayer composed of hydrocarbon chains of the amphiphiles. However, the barrier is not absolute, but instead permeability varies over eight orders of magnitude for different solutes. A good way to understand this is to compare the half times for the exchange of solutes across the membranes of lipid vesicles called liposomes. Small neutral molecules like water, oxygen and carbon dioxide have half times of exchange measured in milliseconds [16] while monovalent cations like sodium and potassium have half times measured in hours [17]. The non-polar phase presents an extremely high Born energy barrier to the free diffusion of monovalent cations like sodium and potassium, and this allows the cellular compartment to maintain ion gradients that are essential as an energy source for nutrient transport and excitability. Typical phospholipid bilayers are also relatively impermeable to amino acids and phosphate [18], so if primitive metabolic pathways in early cellular life involved amino acids and phosphate, as seems likely, some form of membrane transport mechanism was required to provide access to external sources of nutrients.

Certainly the function of membranes as a container is essential to life, but after primitive cells emerged on the early Earth the membranous boundary soon evolved equally important functions that are essential to all life today. Perhaps the most prominent is the role of lipid bilayer membranes in energy transduction. The interior non-polar phase of a bilayer provides a site for pigment molecules such as chlorophyll that could not function in photosynthesis if dispersed in the aqueous phase of cytoplasm. Furthermore, the membrane confines electron transport reactions in a two-dimensional space and allows them to be coupled to proton transport in order to generate proton gradients across the lipid bilayer barrier. As first elucidated by Peter Mitchell [19], proton gradients across membranes are the primary energy source coupled to ATP synthesis, and this system, now referred to as chemiosmosis, must have accompanied the evolution of primitive protocells into the first forms of cellular life.

What properties are required for amphiphilic compounds to assemble into bilayer membranes that are stable and able to provide a permeability barrier? Although there are exceptions to this rule, the amphiphiles of membranes today are primarily a mixture of phospholipids and sterols. The phospholipids typically have two hydrocarbon chains linked by ester or ether bonds to a glycerol molecule. The presence of two hydrocarbon chains allows phospholipids to assemble into bilayers even at extremely low concentrations in the micromolar range. In contrast, single chain lipids such as fatty acids require millimolar concentrations to assemble into bilayers. The third carbon of glycerol in a phospholipid molecule has an ester bond to a phosphate which in turn has any of several hydrophilic groups linked to it, such as choline, ethanolamine, serine or glycerol.

For stability in an aqueous cytosol of a living cell, the hydrocarbon chains must be in the range of 14 to 18 carbons in length. However, saturated hydrocarbon chains that long would “freeze” into gels at ordinary temperature ranges, so metabolic pathways have evolved that add unsaturated *cis* double bonds near the center of the chain [20]. To get a sense of how much effect this has, compare the melting point of saturated stearic acid with 18 carbons (69 °C) with the melting point of oleic acid, also 18 carbons long but with a single *cis* double bond at the 9–10 position (13 °C). To achieve the same degree of fluidity, some microorganisms such as archaea use branched chain lipids in which methyl groups are attached along the length of the chain. The unsaturated bonds and chain branching both increase fluidity by reducing the ability of hydrocarbon chains to pack tightly and form extensive van der Waals interactions.

The sterol component of lipid bilayers in biological membranes is most often cholesterol with four rings, a hydrophobic hydrocarbon tail and a hydrophilic hydroxyl group at the other end of the molecule. Cholesterol has the effect of filling transient defects in fluid bilayers, thereby increasing stability and reducing permeability to ions and polar solutes [21]. This is significant because there is evidence that certain polycyclic aromatic hydrocarbon compounds could fulfill a similar function in primitive membranes [22].

We can now summarize the properties of amphiphilic molecules that compose biological membranes. These are typical, but there are rare exceptions to the rule.

- Phosphate is a primary anionic component of most membrane lipids. Sulfate and carboxylate groups are also present on certain lipids
- Two fatty acids are attached to a glycerol by ester or ether bonds.
- The hydrocarbon chains must be in a fluid state at temperatures ranging from 0 to 100 °C depending on the organism’s environment.
- The hydrocarbon chain lengths must be sufficiently long to be stable as bilayers, and also to maintain a permeability barrier to ionic and polar solutes. Laboratory studies have shown that fatty acids as short as 10 carbons can assemble into fragile membranes, but typical chain lengths of eukaryotic membrane phospholipids are in the range of 16 to 18 carbons.
- Biological membranes are not composed of pure phospholipids, but instead are mixtures of several phospholipid species, often with a sterol admixture such as cholesterol.
- The anionic groups of phospholipids and amino acids strongly interact with divalent cations, particularly calcium (Ca^2+^), so living cells exclude calcium by active outward transport.
- Protons also interact with carboxylate, phosphate and amine groups on lipids and proteins. The protonation state strongly affects lipid properties, so cells maintain intracellular pH near neutrality (pH ~7) by actively pumping protons across their membranes.

Given these properties and conditions, how do they constrain our understanding of membrane-bounded compartments in the prebiotic environment? We will first consider potential sources of abiotic amphiphilic compounds.

## 3. Non-Biological Sources of Amphiphilic Compounds

Anyone who has blown a soap bubble has made a self-assembled membrane. Soaps are monocarboxylic acids, and a soap bubble is a metastable structure with a monolayer of ionic fatty acids on the outside and inside surface. The hydrocarbon chains are directed outward, and the hydrophilic head groups stabilize a thin layer of micelles and water on the interior of the membrane. Soap molecules also assemble into microscopic vesicles in aqueous phases, but the membranes are bilayers with the hydrocarbon chains directed inward. Self-assembly of amphiphilic molecules like soap is so common that it is not difficult to imagine that similar molecules, if available, would form membranous compartments on the prebiotic Earth.

Is it possible that amphiphilic molecules resembling contemporary lipids were available for the first forms of cellular life? This seems unlikely because phospholipids and sterols are products of complex and highly evolved metabolic pathways that incorporate multiple enzyme-catalyzed steps. It is more plausible that the first membranes assembled spontaneously from simpler amphiphilic compounds that were available in the prebiotic environment. Two possible sources are delivery during late accretion, and geochemical synthesis. Although it might seem strange that the first forms of life did not depend on metabolic biosynthesis to have access to membrane-forming lipids, life today still accumulates certain amphiphilic molecules directly from the environment followed by incorporation into membranes. Examples include essential fatty acids such as linoleic and arachidonic acid and the fat soluble vitamins: carotenoids such as vitamin A, the cholesterol derivative vitamin D and a-tocopherol (vitamin E), all of which partition into lipid bilayers to perform their functional role. Certain bacteria, in fact, cannot grow unless fatty acids and cholesterol are supplied as nutrients to be used as components of membrane structure [23]. Table 1 compares properties of biological membrane lipids and lipid-like amphiphiles, some of which could have been components of prebiotic membranes.

Because they are present in carbonaceous meteorites, it is generally accepted that organic compounds such as amino acids and nucleobases can be synthesized by chemical processes in the absence of life. There is also evidence that simpler organic compounds are substantial components of comets, so one possible source of prebiotic organics is delivery during late accretion. This was first suggested by Oro (see [24] for review) and Delsemme [25] then later explored in detail by Chyba and Sagan [26] and more recently reviewed by Ehrenfreund and Cami [27]. Monocarboxylic acids are among the most abundant soluble organic compounds in some, but not all carbonaceous meteorites [28]. However, when one considers the fate of organic compounds delivered during accretion, only a small fraction would survive atmospheric entry. Furthermore, most of what did survive would fall into the ocean and dissolve to produce a very dilute solution. It follows that some sort of concentrating process would be required for life to begin.

## 4. Self-Assembly, Growth and Encapsulation by Lipid Compartments

Although the goal of fabricating synthetic life in the laboratory is not within the scope of this review, a brief description of progress that has been made over the past 50 years will provide a perspective on processes by which cellular life could have originated in natural conditions. The reason that lipids have become so prominent in research on the origin of life is the ease with which they self-assemble into membranous compartments. This property was first discovered Bangham and co-workers in the 1960s [38] and the resulting vesicles soon came to be referred to as liposomes. A few years later Gebicki and Hicks observed that oleic acid could also produce vesicles they called ufasomes [29] and their result was soon extended to a variety of single stranded amphiphiles [30]. A plausible prebiotic process by which macromolecules such as proteins and nucleic acids could be encapsulated was reported a few years later [39]. The next step was to show that enzyme-catalyzed polymerization could occur in vesicles. This was independently demonstrated by Chakrabarti et al. [40] using phospholipid vesicles and in oleic acid vesicles by Luisi and co-workers who went on to perform pioneering studies in which processes such as replication, translation and the PCR reaction were confined within oleic acid vesicles [41,42,43,44,45].

Szostak, Bartel and Luisi wrote a seminal paper in 2001 [46] which proposed that it might now be conceivable to fabricate a synthentic version of life. The Szostak laboratory has made substantial progress toward that goal, which has been reported in a series of papers [47,48,49,50,51,52,53,54,55]. An alternative top down approach was taken by Noireaux and Libchaber, [56] who encapsulated a translation system in lipid vesicles and demonstrated that green fluorescent protein (GFP) could be synthesized and accumulated in the vesicles. This approach was expanded by Yomo and his co-workers [57,58] who were able to establish a membrane-encapsulated genetic cascade involving both transcription and translation. Such investigations clearly define the hurdles that must be overcome for life to begin. For instance, Yomo’s system had no access to external sources of amino acids and energy, so all of the components needed to be present in the lipid vesicle at the start. Noireaux and Libchaber solved this problem by including the gene for hemolysin as well as GFP. Hemolysin assembles into a channel that accommodates both amino acids and ATP as an energy source, so their system had access to nutrients and energy and was able to function for several days.

## 5. Effects of Physical and Chemical Conditions on Self-Assembly Processes

### 5.1. Dissolved Salts and pH Limit Self-Assembly Processes

The effects of dissolved salts on self-assembly must be taken into account when considering the origin of life. For instance, concentration gradients of monovalent salts (NaCl, KCl) across lipid membranes lead to osmotic pressure that can collapse or burst membrane-bounded compartments. Microbial life today can exist because cell walls protect against osmotic disruption, but it seems unlikely that the first forms of cellular life would have cell walls.

Another effect of dissolved salts concerns divalent cations. Anyone who has attempted to use soap in hard water has experienced the problems caused by divalent cations like calcium and magnesium. Instead of the usual detergent effect, the soap molecules aggregate into a curd caused by cations binding tightly to their carboxylate groups. Divalent cations also bind strongly to phosphate in solution, and to phosphate groups attached to organic compounds like nucleotides.

The acidity or alkalinity of an aqueous solution can also have a marked effect on self-assembly. For instance, membranes composed of pure fatty acids are only stable near the pK of the carboxylate group in which there is a mixture of charged and neutral (protonated) head groups. The stabilization results from hydrogen bonding between the head groups, effectively producing the equivalent of transient species having two chains [33]. At low pH ranges where the carboxyl groups are protonated the fatty acid either crystallizes or produces droplets, depending on its melting point, while at high pH ranges in which the carboxyl groups are ionized only micelles are present. It is possible to reduce the effect of pH by adding fatty alcohols or monoglycerides [33] or even cationic alkyl amines [35] but it is uncertain whether any of these would be available in the prebiotic environment.

### 5.2. Temperature Imposes Constraints on Lipid Composition of Membranes

Cells today can exist in temperature ranges from near freezing to near boiling and adjust the lipid composition of their membranes accordingly. In particular, for thermophilic organisms, survival at elevated temperature ranges requires increased stability to both chemical and physical stresses. The archaea, for instance, use ether bonds rather than ester bonds to link hydrocarbon chains to glycerol of phospholipids, presumably because ether linkages are more stable to hydrolysis. Some archaeal membranes are composed of specialized lipids that span the distance from one side to the other, so they exist as monolayers rather than bilayers. Again, this modification markedly increases stability at temperatures near 100 °C and reduces permeability to ionic flux, particularly protons, in order to maintain transmembrane gradients required for bioenergetic functions such as ATP synthesis.

The Fischer-Tropsch type reaction (FTT) is an alternative source of amphiphilic molecules capable of self-assembly into stable membranes at elevated temperatures. The industrial process involves passing carbon monoxide and hydrogen gas over a hot iron catalyst, resulting in a variety of long chain alkanes in good yield. It seems unlikely that such an iron-catalyzed reaction could occur in the prebiotic conditions, but McCollom et al. [59] and Rushdi and Simoneit [60] have shown that a series of carboxylic acids and alcohols are synthesized when formic or oxalic acid solutions are heated under pressure and temperature regimes characteristic of hydrothermal conditions associated with volcanism. Remarkably, when the temperature exceeded 150 °C the oxalic acid decomposed into carbon monoxide and water which then polymerized into hydrocarbon derivatives ranging from 12 to 33 carbons in length. The products included mixtures of normal monocarboxylic acids and alcohols which are well able to self-assemble into membranous vesicles [61].

### 5.3. Stabilization of Bilayers by Admixtures

What do we mean when we state that one lipid bilayer is more or less stable than another? An obvious indication of stability is simply whether a membrane forms at all. For instance, consider dispersions of 50 mM fatty acids. At a pH near the pK, octanoic acid is only present as a clear solution of micelles, while decanoic acid, two carbons longer, is present as a turbid suspension containing both micelles and vesicles bounded by bilayer membranes. As chain length increases, the concentration required to form vesicles decreases. For instance, oleic acid, with 18 carbons and a single *cis* double bond at the 9–10 position begins to form vesicles at a concentration of 3 mM [32]. However, the equivalent chain length without a double bond is stearic acid, which is a solid and unable to assemble into bilayers until the temperature reaches its melting point of 68 °C.

On the other hand, self-assembled membranous vesicles in the prebiotic environment would not be composed of pure compounds, but instead would be mixtures of amphiphiles having varying chain lengths. It is likely that a certain amount of alkanes and polycyclic hydrocarbons would also be components. A significant point is that mixed compositions can be more stable than membranes composed of pure lipids. This was discovered early in the history of membranes when it was found that including alkanes such as tetradecane and hexadecane in phospholipid films improved stability of phospholipid membranes formed across the supporting aperture of a planar bilayer apparatus [62]. In later work, it was observed that addition of long chain alcohols or monoglycerides also had a significant stabilizing effect by reducing the lability of fatty acid membranes to salt and divalent cations [33,34]. Groen et al. [22] found that polycyclic aromatic hydrocarbons tended to stabilize fatty acid membranes in a manner reminiscent of the effect of cholesterol on phospholipid membranes. Significantly, Black et al. [63,64] reported that adenine had a stabilizing effect on fatty acid membranes, suggesting that water soluble nucleobases may also have a role in stabilizing primitive membranes.

### 5.4. Bilayer Membrane Permeability to Ionic Solutes

If the first forms of life were cells, it was essential for internal systems of functional polymers to have access to external supplies of nutrients and energy. From what we know of contemporary cellular life, there are multiple ways to solve this problem ranging from passive diffusion down concentration gradients to energy dependent transport by specialized enzymes embedded in the membrane. The simplest form of transport allows small neutral solutes such as oxygen, carbon dioxide and water to partition into the lipid bilayer and diffuse across. Ions are also small, but the fact that they carry a negative or positive charge decreases their permeability by eight orders of magnitude compared to water. This effect is due to a very large Born energy barrier related to the energy required for an ion to leave an aqueous medium having a dielectric constant of 80 and enter the low dielectric center of the membrane occupied by hydrocarbon chains [65].

Nonetheless, ions do get across at measurable rates because the molecular motions of the lipid molecules composing a fluid membrane are continuously producing transient, hydrated defects in the lipid bilayer that water and ions can enter, thereby avoiding the otherwise nearly impermeable Born energy barrier. Paula et al. [66] showed that the number of defects increased with decreasing membrane thickness, as might be expected. Phospholipids having 14 carbon chains were much leakier to protons and potassium ions that those with 16 and 18 carbon chains, which is the chain length that is optimal for today’s cells living at moderate temperature ranges. This means that the hydrocarbon chains of membranes have lengths that are not so short that the bilayers are unstable, nor too long because they must be fluid rather than gel phases. The membranes of the first cells presumably required a similar optimal chain length in order to function.

## 6. Solving the Problems of Salt and Concentration: Origin of Life in Fresh Water Pools

**Figure 2:**
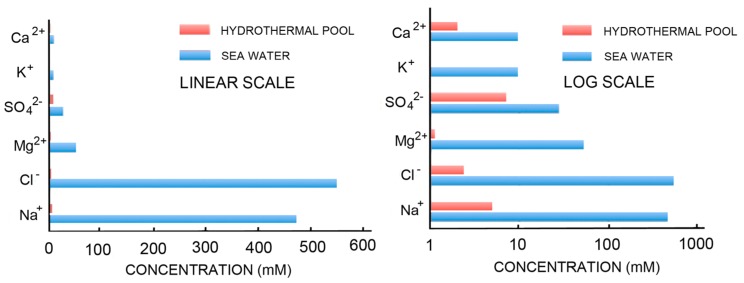
Ionic composition of seawater and fresh water hydrothermal fields.

The simplest way to avoid the problems associated with concentrating potential reactants and inhibition of self-assembly by the divalent cations of seawater is to consider that the first cells may have originated in fresh water associated with volcanic land masses. Figure 2 compares the ionic components of seawater and hydrothermal fields [67] on both linear and log scales. The ionic solutes of hydrothermal water barely register on the linear scale that is dominated by the salt composition of seawater. The log scale shows hydrothermal ionic concentrations in the millimolar range while seawater today is 0.6 M Na^+^, 54 mM Mg^2+^ and 10 mM Ca^2+^.

**Figure 3:**
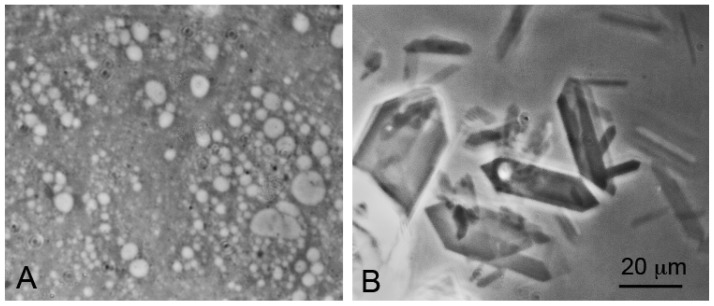
Effect of seawater on self-assembly of lipid bilayer membranes. (**A**) Vesicles composed of decanoic acid readily form in dilute ionic solutions at pH 7.2; (**B**) In seawater at the same pH, crystals of a calcium-magnesium decanoate form instead of vesicles, thereby preventing the self-assembly of bilayer membranes. Bar shows 20 μm in both images.

Self-assembly of anionic amphiphiles into membranous vesicles is strongly inhibited by the divalent cation concentrations in this range but readily occurs in dilute ionic concentrations. Figure 3 shows fatty acid membranes composed of 50 mM decanoic acid at pH 8.3 in a 20-fold dilution of sea water (25 mM NaCl, 2.7 mM MgCl_2_ and 0.5 mM CaCl_2_, Figure 3A) and in undiluted sea water (Figure 3B). Decanoic acid readily forms membranous vesicles in the diluted seawater simulating the ionic composition that might be present in a hydrothermal pool. However, in seawater the divalent cations present cause the decanoic acid to precipitate as crystals and aggregates of Ca^2+^/Mg^2+^ decanoate, making it impossible for membranes to form. Although fatty acids are strongly affected by divalent cations, amphiphiles such as alcohols and monoglycerides do not interact with cations. Monnard et al. [68] showed that adding these as components allowed membranous vesicles to form in seawater.

The 0.6 M NaCl (1.2 OsM) in seawater can also inhibit the self-assembly of phospholipids into vesicles because of the strong osmotic effect that reduces the ability of the phospholipid to swell by taking up water. The assembly of any membranous compartment in seawater will be subjected to this osmotic effect which can be balanced only if the interior concentration of salt balances the external concentration. It may be significant that no living cell today has an intracellular concentration of 0.6 M NaCl. Instead, most cells use active transport to maintain the internal concentration of KCl at approximately 0.1 M.

**Figure 4:**
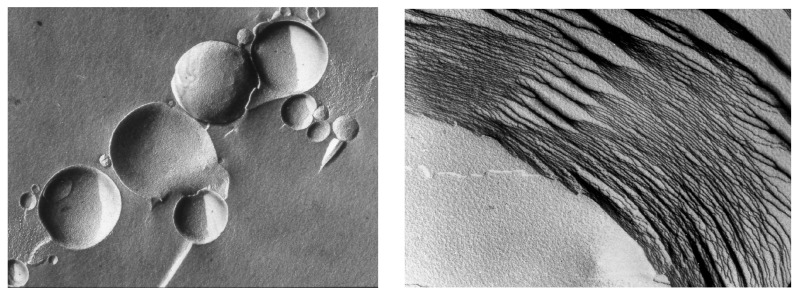
Vesicular membranes 1–10 μm in diameter self-assemble from amphiphilic molecules like phospholipids, as shown in the freeze-fracture image (**left panel**). When dried, the vesicles fuse and flatten to form multilamellar structures (**right panel**). Each of the layers is the dimension of a single lipid bilayer ~5 nm thick. In the freeze-fracture method, lipid bilayers are split within the plane of the hydrocarbon chains, not along the head groups.

The other favorable quality of hydrothermal field sites is that they are fluctuating environments undergoing continuous cycles of hydration and dehydration. As small pools dehydrate by evaporation, any organic compounds become concentrated as films on mineral surfaces, thereby promoting reactions that cannot occur in dilute solutions. Dehydration also serves to promote encapsulation. When amphiphilic compounds are dried in the presence of organic solutes and their polymers, they fuse into multilamellar matrices (Figure 4). Earlier studies demonstrated that water soluble compounds present in the solution *outsid*e of the vesicles are encapsulated within layers of the multilamellar phase produced by drying [38,39].

**Figure 5:**
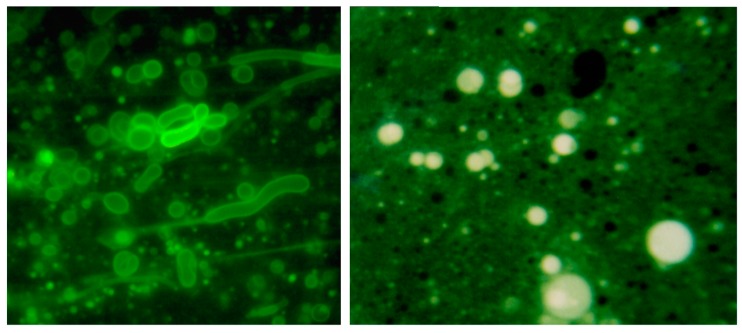
Membranous vesicles self-assemble from mixtures of fatty acids and alcohols such as decanoic acid and 1-decanol [61]. The vesicles shown here (**left panel**) were stained with rhodamine 6G and photographed by fluorescence microscopy (400× original magnification) [68]. Typical vesicles shown in the micrograph are ~1–10 μm in diameter. After a single dehydration cycle (**right panel**), the vesicles readily encapsulated macromolecules such as ~600 nt duplex DNA. The DNA was stained with acridine orange, an intercalating fluorescent dye.

For instance, Figure 5 shows short strands of DNA that have been dehydrated in the presence of a 1:1 mixture of decanoic acid and decanol. This mixture readily assembles into bilayer membranes, and the encapsulated DNA fluorescently labeled with acridine orange dye can be seen in numerous vesicles. Protocells can be considered to be microscopic experiments in a natural version of combinatorial chemistry.

## 7. Condensation Reactions and Polymerization

In life today, polymerization occurs in the aqueous cytoplasm of cells, with ribosomes synthesizing proteins and a variety of polymerases synthesizing nucleic acids. The linking bonds of these polymers are peptide and ester bonds. In both cases, the polymerization reaction is thermodynamically uphill, with hydrolysis being favored. How then can polymers be synthesized? The answer, of course, is that the monomers have been chemically activated by an input of metabolic energy so that polymerization is spontaneous in the presence of the enzymes or ribosomes that catalyze polymerization. The substrate for polymerases that synthesize nucleic acids are nucleoside triphosphates, and for ribosomes the substrates are amino acids linked to tRNA.

A plausible mechanism for synthesis of peptide bonds and ester bonds on the prebiotic Earth continues to be a major gap in our understanding of the origin of life. In order to make clear the underlying physical and chemical processes that are relevant to this question, we first need to address the primary hurdles that need to be overcome if non-biological nucleic acid synthesis can be shown to be plausible in the prebiotic environment. Although a source of mononucleotides is still debatable, we will assume that they or similar compounds are present, perhaps by a synthetic pathway similar to that described by Powner et al. [69]. Given a source of ordinary mononucleotides, several obvious questions immediately come to mind:

1. How can the monomers be sufficiently concentrated and organized to promote polymerization?
2. What is the source of chemical potential to drive condensation reactions?
3. How can polymers accumulate even though hydrolysis is thermodynamically favored?

One approach to answering these questions involves an axiom of thermodynamics: All reactions proceed toward equilibrium, and all reactions are in principle reversible. We also rely on one of the first principles of kinetics, that products will accumulate in a kinetic trap if a synthetic reaction such as anhydrous condensation to form ester bonds has a rate constant that exceeds that of hydrolysis that occurs in the presence of water. All life, in fact, exists in a kinetic trap. Energy dependent polymer synthesis is faster than spontaneous polymer hydrolysis, and therefore polymers and monomers such as nucleic acids and mononucleotides are not at thermodynamic equilibrium, but instead exist in a steady state maintained by an input of chemical energy.

A reasonable assumption is that hydrothermal fields associated with volcanic activity are analogues of the prebiotic environment [9]. Hydrothermal fields have small acidic pools of fresh water heated by geothermal energy [8] with characteristic cycles of evaporation and filling. These conditions can be simulated in the laboratory, and it is possible for dehydration to lower water activity to the point that water molecules become leaving groups. If mononucleotides are present, they become sequestered in a fluid multilamellar matrix that promotes condensation by concentrating and organizing the monomers, thereby overcoming entropy barriers that would otherwise inhibit polymerization [70].

### Phosphodiester Bond Synthesis

Fluctuating environments in the form of wet-dry cycles at elevated temperature ranges have long been considered to be possible sources of free energy that could drive uphill polymerization reactions. For instance, Usher [71] proposed that cycles of heating and drying, followed by rehydration, could drive phosphodiester bond formation and promote the accumulation of 3′-5′ bonds in the system due to the relative lability of 2′-5′ bonds to hydrolysis. Verlander et al. [72] showed that anhydrous heating of mononucleotides could drive the formation of mixed 2′-5′ and 3′-5′ phosphodiester bonds. Lohrmann and Orgel [73] also explored this possibility, but were only able to achieve dimers and trimers in low yields. Pioneering studies were also carried out by Lahav and White [74] who investigated polymerization of amino acids and nucleotides by drying the reactants on clay mineral surfaces.

In contrast to the conditions above in which mononucleotides were simply dried, Burcar et al. [75] explored the possibility that phosphoester bonds might form in a laboratory simulation of hydrothermal vent conditions and reported that small yields of dinucleotides could be detected when non-activated ribonucleotides circulated through an iron-sulfur rich chimney. Although anhydrous conditions resulting from drying also lead to dimers and trimers, when monomers are dried the potential reactants are disorganized and immobilized within the solid matrix of a bulk phase so that reactive groups only rarely come into contact to undergo condensation reactions. However, if a microenvironment not only organized the mononucleotides but also permitted diffusional mobility, it is possible that longer oligonucleotides resembling RNA would be synthesized from monomers.

**Figure 6:**
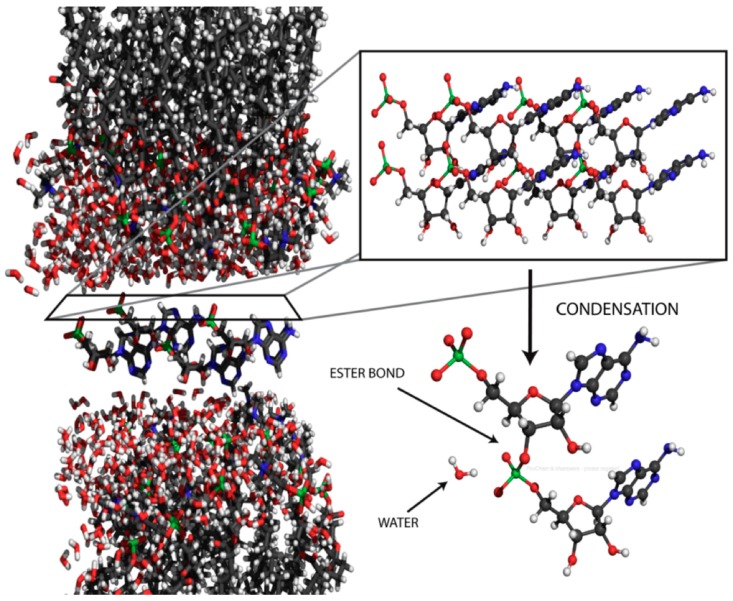
X-ray diffraction structure of a lamellar lipid matrix that has imposed order on a solute, in this case 5′-adenosine monophosphate [76].

One such condition is that monomers can be concentrated within highly ordered multilamellar phases sometimes referred to as liquid crystals. Toppozini et al. [76] used X-ray diffraction to establish that ordering of a mononucleotide within the two dimensional planes of a multilamellar lattice does in fact occur (Figure 6). If solutes are present in a solution of amphiphiles during dehydration, the solutes become highly concentrated and organized within a two-dimensional matrix. The order imposed on the solutes by the liquid crystalline matrix allows them to undergo polymerization reactions that cannot occur in dry bulk phases or in dilute aqueous solutions.

A working hypothesis is that ester bond synthesis can be driven in concentrated mononucleotides when water activity is decreased during dehydration. The hypothesis was tested by De Guzman et al. [77] and Da Silva et al. [78] who investigated RNA synthesis at elevated temperature ranges in fluctuating environments simulating hydrothermal springs and ponds that were likely to have been common in the prebiotic Earth. De Guzman et al. used lysophosphatidylcholine as an organizing agent with mixtures of 5′-AMP and 5′-UMP as monomers, while Da Silva et al. used monovalent salts: LiCl, NaCl, KCl and NH_4_Cl, with the same mixture of mononucleotides. Both approaches produce polymers with the following properties:

1. The products could be isolated by ethanol precipitation or spin tubes designed to bind RNA.
2. The products could be separated by gel electrophoresis and either labeled with radioactive phosphate or stained with intercalating dyes such as ethidium bromide. They exhibited chain lengths from 10 to 50 nucleotides in the first few cycles, and in later cycles the chain lengths exceeded 100 nucleotides.
3. The products exhibited hyperchromicity, suggesting that intramolecular duplex structures such as hairpins were present.
4. When analyzed with a hemolysin nanopore apparatus, the products produced ionic current blockades similar to those expected of single stranded RNA.

**Figure 7:**
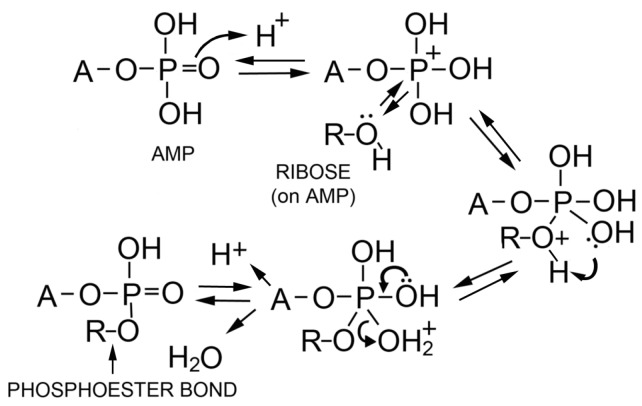
Proposed mechanism for phosphoester bond formation in a condensation reaction driven by simulated hydrothermal conditions [79].

The authors concluded that polymers resembling nucleic acid oligomers are synthesized by wet-dry cycles as long as they were dehydrated in the presence of a promoter such as multilamellar liquid crystals or eutectic mixtures associated with crystallizing monovalent salts. Although the precise mechanism is not yet understood, one possibility is that at low pH a phosphoryl oxygen (P=O) group on the phosphate becomes protonated to -OH^+^ which then passes through several intermediate states shown in Figure 7. The 2′ or 3′ hydroxyl of a neighboring ribose then can undergo a nucleophilic attack on the phosphorus to produce an ester bond when a water molecule becomes a leaving group. The moderately elevated temperature of 60–90 °C provides activation energy for the reaction without significant degradation of reactants or products. Significantly, if amphiphilic compounds are present during dehydration, the polymer products that are synthesized become encapsulated upon rehydration.

## 8. Decomposition of Monomers, Polymers and Molecular Systems: An Unresolved Problem

From the results described above, it is clear that non-activated nucleotide monomers can be linked into polymers under certain laboratory conditions designed to simulate hydrothermal fields. However, both monomers and polymers can undergo a variety of decomposition reactions that must be taken into account because biologically relevant molecules would undergo similar decomposition processes in the prebiotic environment.

Hydrolysis of ester and peptide bonds has already been described above as a spontaneous reaction that must be compensated in some way by condensation reactions. However, there are two other reactions that can affect mononucleotides as monomers of nucleic acids. *Depurination* occurs when the glycoside bond is broken that links purines (adenine and guanine) to a ribose or deoxyribose either in a mononucleotide or a nucleic acid. This hydrolysis reaction is spontaneous and occurs continuously in the DNA of all genomes. In a living cell, abasic sites left by depurination are quickly repaired by the action of specific enzymes, but in prebiotic conditions adenine and guanine would be lost at a certain rate from any nucleotide or nucleic acid. Another spontaneous reaction is the *deamination* of cytosine to produce uracil. This was recognized by Shapiro [80] as an important problem related to the origin of a genetic code.

Carbohydrates are also subject to chemical damage. For instance, amine groups can react with ribose and other sugars to produce cross linking in the Maillard reaction. Reducing sugars can also react with other sugar molecules at higher temperatures, a process called caramelization. In both cases this produces the familiar brown polymer present in all baked or grilled food, but would interfere with the synthesis of biologically relevant bonds.

*Undesired crosslinking reactions.* At ordinary temperature ranges in aqueous solution there is insufficient activation energy to drive random crosslinking between biologically relevant monomers, and the thermodynamically favored decomposition reaction is hydrolysis. When solutions are dehydrated by evaporation, solutes become concentrated, and in the anhydrous state the thermodynamic balance shifts from hydrolysis to the condensation reactions described earlier. As the temperature increases up to 100 °C increasing activation energy becomes available to promote the synthesis of low energy linking bonds so that ester and peptide bonds can produce oligomers. However, if the temperature increases further from 150 to 300 °C, the activation energy becomes sufficient to break high energy bonds between carbon, nitrogen and oxygen so that pyrolysis become a dominant reaction, accompanied by synthesis of highly stable polymers referred to as kerogen and tar. Benner et al. [81] pointed out that such products are unable to participate in biologically relevant processes. However, tars are defined as viscous black fluids produced by destructive pyrolysis and asphalts are solid polymers produced from biological hydrocarbon derivatives by the high temperatures and pressures associated with deep geological formations. Synthesis of tars and asphalts requires temperatures and activation energies high enough to form extensive carbon-carbon bonds that lead to polymerization. In contrast, the bonds linking biopolymers are simple ester, peptide and glycoside bonds that have low activation energy and can be synthesized or broken by hydrolysis at ordinary pressure and temperatures below the boiling point of water. There is no evidence of tar or asphalt formation in hydrothermal fields. Instead, the primary degradation reactions are hydrolysis of polymers and depurination of both polymers and oligomers.

*Osmotic disruption of protocells.* From early studies of liposomes it became apparent that vesicles bounded by lipid bilayer membranes responded to osmotic pressure generated by concentration gradients [38]. Osmotic effects cause two potential problems for early cellular life. High concentrations of osmotically active salts inhibit the ability of membranous vesicles to form because the internal volume cannot increase unless the membrane is sufficiently permeable for the salt to equilibrate between the internal and external phases. The other problem occurs if the external medium becomes diluted. As a result, the compartment swells as water diffuses inward across the boundary membrane and may even burst, releasing the internal contents. This is precisely what occurs when red blood cells are placed in a hypotonic medium, a process called hemolysis.

Although decomposition reactions are inevitable, there must have been processes by which the earliest forms of life overcame such damage in order to emerge out of a complex mixture of organic and inorganic solutes. There are at least three possible solutions. The simplest, still a feature of life today, is to synthesize polymers faster than they are degraded by hydrolysis. This allows the polymers of life to exist in a steady state in which hydrolysis is under the control of specialized enzymes such as proteases, nucleases, lipases and others. It is reasonable to think that the earliest forms of cellular life also survived by establishing a similar steady state condition.

## 9. Future Research Directions

The question of how life began is like a jigsaw puzzle with the pieces in a jumble, some right side up and others upside down. Using what we know of chemistry, physics and thermodynamics, previous research has assembled a frame of pieces around the outside of the puzzle, but there are still huge gaps in the center. The discussion presented here as well as in a recent review by Tessera [79] have offered several insights on how to fit together the missing pieces. I will finish by posing a list of questions related to the role of lipids in the origin of life, with the hope that they will guide and inspire the next generation of scientists to try to answer them.

*1.* *What is a plausible source of amphiphiles on the early Earth?*

It is reasonable to assume that simple amphiphiles were available in the prebiotic inventory, but sources of compounds with hydrocarbon chains sufficiently long to form stable membranes are still uncertain.

*2.* *What concentrating mechanisms could occur in conditions related to hydrothermal fields?*

In fluctuating pools of hydrothermal fields, concentration occurs naturally by evaporation, but as yet no process has been tested by which organic compounds can be transported to the pools.

*3.* *How can membranous vesicles assemble in complex mixtures of organic compounds and ionic solutes?*

It is essential to establish mixtures of amphiphilic compounds that can assemble into stable membranous vesicles in the face of variables such as elevated temperatures, acidic and alkaline pH ranges, osmotic effects of monovalent salts, and instability caused by the presence of divalent cations.

*4.* *What lipid compositions can provide a membrane barrier sufficient to maintain proton gradients?*

If chemiosmosis was used by primitive cells as an energy source, there must have been lipid mixtures available that could assemble into membranes capable of maintaining a proton gradient. Phospholipids with two hydrocarbon chains are up to this task, but can simpler mixtures of single-chain amphiphiles provide a barrier to proton permeation?

*5.* *How confident can we be that laboratory simulations accruately reflect reactions and self-assembly processes that could occur in the prebiotic environment?*

We often claim that the reactions investigated in the laboratory using purified water, buffered pH, and pure reagents could also occur in the prebiotic environment, but this confidence may be unwarranted. Hydrothermal fields are abundant in volcanic sites such as Kamchatka, Iceland, New Zealand, Hawaii and the volcanoes associated with subduction zones of the Pacific Rim. We should be bold enough to rise to the challenge and attempt to show that processes simulated in the laboratory actually work in such prebiotic analogue environments.

## Figures and Tables

**Table 1:** Comparison of contemporary membrane lipids and candidates for prebiotic self-assembled membranes.

*Biological membrane lipids*
Source: enzyme-catalyzed synthesis during metabolism
Phospholipids with ester or ether bonds linking fatty acids or alcohols to glycerol
Two hydrocarbon chains, typically 16–18 carbons, with unsaturation or branching
Admixture of cholesterol helps to stabilize the bilayer
Functions of lipid bilayers: Fluid barrier to free diffusion of ions, nutrient solutes, metabolites Site of pigments involved in photosynthesis Site of electron and proton transport enzymes
*Examples (not phospholipids) of amphiphilic compounds and mixtures that form stable membranes at ordinary temperature ranges*
Fatty acids [29,30,31,32]
Mixtures of fatty acids and fatty alcohols [33]
Mixtures of fatty acids and monoglycerides [34]
Mixtures of fatty acids and alkylamines [35]
Mixtures of fatty acids and PAH [22]
Sodium dodecyl sulfate and dodecyl alcohol [30]
Chlorosulfolipids [36,37]
